# Empirical model of teachers’ neuroplasticity knowledge, mindset, and epistemological belief system

**DOI:** 10.3389/fpsyg.2022.1042891

**Published:** 2022-12-08

**Authors:** Khalil Gholami, Maryam Alikhani, Kirsi Tirri

**Affiliations:** ^1^Department of Education, Faculty of Humanities and Social Sciences, University of Kurdistan, Sanandaj, Iran; ^2^Department of Education, Faculty of Educational Sciences, University of Helsinki, Helsinki, Finland

**Keywords:** neuroplasticity, teachers’ mindset, epistemological belief system, teachers’ knowledge, neuromyths, educational neuroscience

## Abstract

Educational research has shown that teachers’ knowledge and beliefs are two important variables that significantly affect their pedagogical practice and decisions. Relying on the premise that knowledge is superior to beliefs in a pure epistemic dimension and rooted in the previous empirical studies, we examined the hypothesis that teachers’ knowledge of neuroplasticity affects their epistemological belief system mediated by mindset. Using a survey consisting of established scales about these variables, we collected data from a sample of 345 teachers. Structural equation modeling was performed to test the hypothesis. Results showed that the path coefficients (direct effects) from teachers’ knowledge of neuroplasticity to their mindset and epistemological belief system were statistically significant. In other words, we found that teachers with a higher score in the knowledge of neuroplasticity had a growth mindset and a sophisticated epistemological belief system. Teachers’ knowledge of neuroplasticity also had an indirect effect on their epistemological belief system mediated by mindset. This result has a conceptual contribution to the literature because it suggests that teachers’ knowledge of neuroplasticity is a predicting variable for mindset and epistemological belief system. In practice, it provides us with a tool for developing teachers’ growth mindset and sophisticated epistemological beliefs.

## Introduction

This paper is based on the premise that teachers’ knowledge of educational neuroscience dispels their naïve epistemological belief systems and fixed implicit theories on intelligence. Many teachers have acquired what Bruner (1996, p. 46) calls “folk pedagogy,” which reflects certain “wired-in human tendencies” and some deeply fixed beliefs rooted in their social and personal experiences that lack scientific evidence. Empirical research suggests that a significant part of such folk pedagogy is the prevalence of misconceptions about the brain, which are called “neuromyths,” among teachers in different countries and various educational settings (Howard-Jones, 2014; Gleichgerrcht et al., 2015; Dündar and Gündüz, 2016; Ferrero et al., 2016; Düvel et al., 2017; Blanchette Sarrasin et al., 2019; Carter et al., 2020; Torrijos-Muelas et al., 2021; Jeyavel et al., 2022). In 2002, the Brain and Learning project of the Organization for Economic Co-operation and Development (OECD) warned that the rapid proliferation of neuromyths among teachers and other professionals is a challenging phenomenon in educational settings (OECD, 2002). In a comparative study among teachers in the United Kingdom and Netherlands, Dekker et al. (2012) found that, on average, teachers believed 49% of the neuromyths. However, research has provided evidence against such neuromyths, such as left vs. right brain people, only 10% of brain use, multiple intelligences, and visual, auditory, and kinesthetic (VAK) learning styles (Torrijos-Muelas et al., 2021).

Holding a personal belief or relying on knowledge to make pedagogical choices is the matter of warrant by which teachers justify their actions. Adapting from Freeman, there could be four types of warrants in teaching: *a priori* warrant that involves resorting to a pedagogical or scientific principle; an institutional warrant is a justification of a pedagogical choice on the grounds of it being recommended or required in a textbook (institutional–curricular); an empirical warrant is the citation of a frequent occurrence in the classroom or the resorting to personal learning experiences; and an evaluative warrant is a justification of a pedagogical choice on the grounds of a personally held view, value or belief (Nardi et al., 2012). In this research, teachers’ knowledge of neuroplasticity relies on *a priori* warrant and teachers’ beliefs may be supported by empirical and evaluative warrants. Educators and policymakers need to plan for promoting teachers’ knowledge of the brain or educational neuroscience to dispel neuromyths among teachers and thus ground their pedagogical beliefs on priori warrant. Dekker and Jolles (2015) state that “learning about the brain and neuropsychological development in adolescents may increase teachers’ understanding of typical adolescent behavior such as risk taking…. This may positively influence teachers’ patience and optimism, as well as help them to develop an effective professional attitude toward students” (p.1). Other empirical research suggests that teachers’ knowledge of educational neuroscience significantly reduces their neuromyth beliefs (Wilcox et al., 2021; Ferreira and Rodríguez, 2022); improves the quality of learning, and promotes equity among learners (Coch, 2018); enhances educators’ pedagogical practice and thinking to meet learners’ diverse needs (Walker et al., 2019); provides teachers a platform to promote students’ motivation and engagement (Dubinsky et al., 2019); and develops teachers’ pedagogical practice, enhances stronger relationships between teachers and learners, and increases meaningful learning (Hachem et al., 2022). A significant part of teachers’ folk pedagogy and naïve pedagogical beliefs root in the lack of scientific knowledge about relevant phenomena they deal with in the teaching-learning process. In other words, when teachers have no knowledge about something, there is a strong possibility to grasp false beliefs about it. In line with this concern, we examined the empirical relationship between teachers’ knowledge of neuroplasticity, teachers’ theories of intelligence or mindset, and teachers’ epistemological belief system and posed the following research questions:

1. To what extent does teachers’ neuroplasticity knowledge affect their epistemological belief system and mindset?
2. To what extent does teachers’ mindset affect their epistemological belief system and mediate the relationship between teachers’ neuroplasticity knowledge and their epistemological belief systems?

## Definition of the main variables

Generally, neuroplasticity “refers to the capacity of neurons and neural networks to change their connections and behavior in response to experience” (Dan, 2019, p. 1). “Plasticity embodies the idea that the strength of the synaptic connections between neurons is dynamic, becoming stronger with the use or weaker with inactivity…synchronous plasticity in the neural pathways producing specific behaviors results in observable learning” (Dubinsky et al., 2013, p. 318). In the educational context, particularly in schools, teachers’ neuroplasticity knowledge has important implications for their pedagogical practice and beliefs toward students’ learning. As such, neuroplasticity has been one of the main theme of research in educational neurosciences for teachers’ professional development programs (Hachem et al., 2022).

Mindset is defined as “implicit theories about the malleability and stability of human characteristics related to ability, intelligence, and talent” (DeLuca et al., 2019, p. 159). According to Dweck (2007), mindset consists of believing that personal characteristics are either entirely malleable (growth mindset) and thus can be developed or entirely fixed and unchangeable (fixed mindset; see Dweck, 1999; Yeager and Dweck, 2020). Students with a fixed mindset “reject opportunities to learn if they might make mistakes, afraid of effort because effort makes them feel dumb and do not recover well from setbacks” (Dweck, 2007, p. 2). By contrast, “students with a growth mindset seek challenges, rebound from failures, and accept feedback for improvement” (DeLuca et al., 2019, p. 159). There has been increasing interest among educational researchers to examine how teachers’ and students’ mindsets relate to their practice, beliefs, and other important functions.

Rooted in the theory of personal epistemology, Schommer-Aikins (2004) introduced and defined the concept of epistemological belief system as a system of independent beliefs about “(a) the stability of knowledge, ranging from unchanging knowledge to tentative knowledge; (b) the structure of knowledge, ranging from isolated bits and pieces to integrated concepts; (c) the source of knowledge, ranging from omniscient authority to reason and empirical evidence; (d) the speed of learning, ranging from quick or not-at-all to gradual; and (e) the ability to learn, ranging from fixed at birth to improvable” (p.20). In this way, an individual may hold more than one sophisticated or naïve belief system over a continuum considering different dimensions of the epistemological belief system (Schommer, 1990, 1993). For example, a person may have highly sophisticated beliefs about speeds of learning but a naïve belief about the source of knowledge.

## Research conceptual framework and hypotheses

In the present research, considering the main variables, we have formulated four hypotheses. Empirical research has suggested that teaching neuroplasticity in an educational setting induces a growth mindset about motivation, goals, effort beliefs, response to failure, and academic enjoyment (Sarrasin et al., 2018). “If teachers know that the underlying brain networks for planning abilities continue to mature during adolescence and that this development is contingent upon experiences, they will understand that they have to provide more guidance to stimulate the development of students’ planning abilities” (Dekker and Jolles, 2015, p. 2). In addition, in teaching studies, researchers are interested in teachers’ epistemological belief system and the ways they are related to their pedagogical practices and personal characteristics (Sinatra and Kardash, 2004; Jones and Carter, 2006; Bernardo, 2008; Yilmaz-Tuzun and Topcu, 2008; Topcu, 2013; Bahçivan, 2016; Demirbag and Bahcivan, 2022). In general, sophisticated epistemological belief system enable pre−/in-service science teachers to gain more constructivist perspectives on learning and teaching (Demirbag and Bahcivan, 2022). In most previous studies, both teachers’ epistemological belief systems and neuroplasticity knowledge were examined as predicting variables for teachers’ pedagogical thinking and practice. We argue that teachers’ knowledge of neuroplasticity has however a more concrete epistemic position compared to the epistemological belief system and mindset; thus, we used it as the main predicting variable for teachers’ epistemological belief system and mindset. As such, two hypotheses examine the direct effect of teachers’ knowledge of neuroplasticity on their epistemological belief system and mindset:

*Hypothesis 1*: Teachers with correct knowledge of neuroplasticity hold less likely a naïve epistemological belief system.

*Hypothesis 2*: Teachers with correct knowledge of neuroplasticity have less likely a fixed mindset.

Considering mindset, the results of several studies have found that mindset has a significant effect on students’ characteristics such as academic achievement, motivation, and effort beliefs (Blackwell et al., 2007); entrepreneurial self-efficacy and career development (Burnette et al., 2020); metacognitive skills on math engagement (Wang et al., 2021); IQ and personality mindset beliefs (Orosz et al., 2017); and stereotype threats about their capabilities (Aronson et al., 2002; Good et al., 2003). In general, the results of these studies have found that students “who hold more of a growth mindset are more likely to thrive in the face of difficulty and continue to improve, while those who hold more of a fixed mindset may shy away from challenges or fail to meet their potential” (Yeager and Dweck, 2020, p. 1; see Dweck and Yeager, 2019). Another major tendency in research on mindset focuses on how teachers’ mindset is presented in their pedagogical practices and how that can be integrated into teacher education programs (Rissanen et al., 2018a,b, 2019, 2021; DeLuca et al., 2019). The results of these studies suggest that teachers’ mindsets “influence their ways of interpreting students’ behavior, learning, and achievements, which in turn guide teachers’ pedagogical thinking as well as their practices for motivating the students” (Rissanen et al., 2018a, p. 487). Generally, teachers with a growth mindset tend to engage in a more advanced, flexible, and moral practice while teachers with a fixed mindset tend to engage “in prescriptive and closed-ended tasks with less descriptive feedback” (DeLuca et al., 2019, p. 160). Therefore, in the previous research, the mindset has been mainly used as a predicting variable for students’ and teachers’ characteristics. In this research, we used mindset as a mediating variable that alters the relationship between teachers’ knowledge of neuroplasticity and their epistemological belief system. Therefore, two more hypotheses were posed as follows:

*Hypothesis 3*: Teachers with a growth mindset hold more likely a sophisticated belief system.

*Hypothesis 4*: Teachers’ mindset mediates the negative relationship between teachers’ knowledge of neuroplasticity and their epistemological belief system.

**Figure 1:**
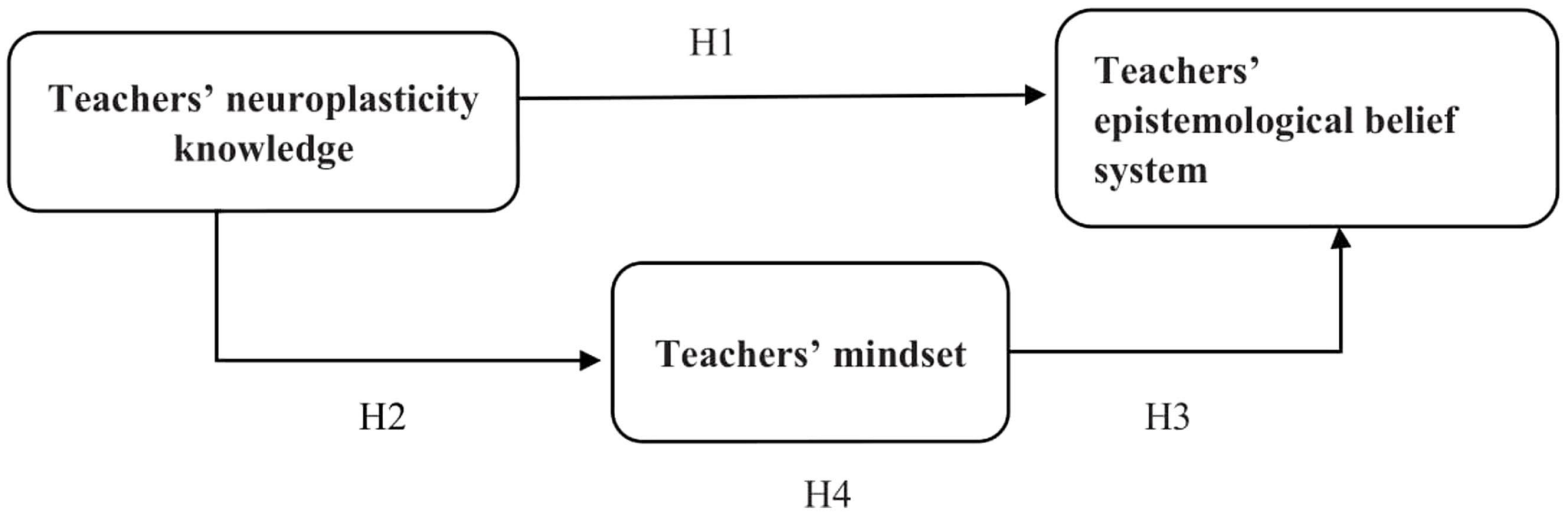
The conceptual model of relationships between the main variables of the study.

Considering these research hypotheses and based on the previous studies, we developed and tested the following research conceptual model (Figure 1).

## Materials and methods

### Participants

A total sample of 345 teachers from Sanandaj, the capital city of the Kurdistan province of Iran, participated in the present research. The total number of teachers in this region was around 3,000, and the sample size was proportional to its population (Krejcie and Morgan, 1970). The participants were in-service subject (35.9%) and pre-service class (64.1%) teachers. The other teachers’ demographic data included gender (female = 30.04%; male = 69.6%), age (18–20 years old = 16.5%, 21–35 = 59.7%, 36 and older = 23.8%), and teaching experiences (pre-service teacher = 64.1%, 1–5 years = 8.7%, 6–10 years = 3.8%, 11–20 years = 9.9%, and 21 years and more = 13.6%). We studied the effects of these demographic data to make sure that the empirical relationship between the main variables is reliable (see the results). The participants were from public schools and participated in the study voluntarily.

### Procedure

First, official permissions were granted from the selected public schools and teacher education universities (in Iran called Farhangian University) to enter the sites for data collection. Second, for in-service teachers, one of the researchers approached the teachers in the selected schools, explained the aim of the research, and asked them to participate in the study voluntarily. For trainee teachers, the researcher and one of the authorities from Farhangian University approached the students while they were in class. Permission from the teacher educators had already been obtained to enter the classes for collecting data. Third, the volunteer teachers were provided a paper questionnaire, and they filled in the questionnaires and returned them to the researchers the same day.

### Measures

The survey consisted of four sections. In the first part, participants provided demographic data, including age, gender, years of teaching, and the subject of teaching. The second part consisted of 18 statements about the brain (Dekker et al., 2012). In this paper, we analyzed nine statements that aim at measuring the knowledge of neuroplasticity (Appendix). In the third part, we used six statements from Dweck’s scale that measures mindset about intelligence and giftedness (Dweck, 2006). Three statements were about mindset on intelligence, and three statements measured mindset on giftedness. In our previous research, we used this scale, and it had strong construct validity and reliability (Rissanen et al., 2018b; Zhang et al., 2019). The fourth part consisted of 24 statements about the epistemological belief system chosen by Schommer (1998). We used the second-order constructs including four dimensions of the epistemological belief system, namely fixed learning ability, simple knowledge, quick learning, and certain knowledge. Fixed learning ability (items 1–6), statements that measure ability to learn ranging from the belief that the ability to learn is fixed to the belief that it can be improved. Simple knowledge (items 7–13), is statements that measure the structure of knowledge as isolated or highly interrelated pieces. Quick learning (items 14–18), statements that measure the speed of learning, ranging from a belief that learning is quick or all-or-none to a belief that it is gradual. Certain knowledge (items 19–24), is statements that measure the nature of knowledge, ranging from a belief that knowledge is certain to the belief that it is evolving.

For all measures, the answer options were “totally disagree,” “disagree,” “agree,” and “totally agree,” which coded 4 for totally agree, 3 for agree, 2 for disagree, and 1 for totally disagree. When entering data in Statistical Package for Social Sciences (SPSS), the following items were reverse-coded: for neuroplasticity, the incorrect items; for mindset, growth items; and for epistemological belief system, sophisticated items. In this way, the higher scores reflect good knowledge, fixed mindset, and naïve beliefs; and the lower scores reflect poor knowledge, growth mindset, and sophisticated beliefs for teachers’ knowledge of neuroplasticity, mindset, and epistemological belief system, respectively.

Using confirmatory factor analysis and Cronbach alpha, we examined the construct validity and reliability of the measures. The factor loading of item 1 for mindset and items 6 and 8 for neuroplasticity did not exceed the cutoff value of 0.5 (Hair et al., 2006) and was removed from further analysis (Table 1).

**Table 1:** Factor loading for the main variables in the study.

Indicators	Epistemological personal beliefs	Neuroplasticity	Mindset
Fixed learning	0.78		
Simple knowledge	0.77		
Quick learning	0.87		
Certain knowledge	0.64		
(1) Learning occurs through the modification of the brain’s neural connections		0.55	
(2) Extended rehearsal of some mental processes can change the shape and structure of some parts of the brain		0.56	
(3) Mental capacity is hereditary and cannot be changed by the environment or experience		0.60	
(4)There are sensitive periods in childhood when it is easier to learn things		0.63	
(5) Learning problems associated with developmental differences in brain function cannot be remediated by education		0.50	
(7) Normal development of the human brain involves the birth and death of brain cells.		0.65	
(9) Vigorous exercise can improve mental function		0.50	
(2) No matter how much intelligence students have, they can always change it quite a bit.			0.62
(3) Students may learn new things, but they cannot change their intelligence.			0.79
(4) Students have a certain talent in certain subjects (e.g., math, sports), and they cannot change it.			0.72
(5) Students can learn new things, but they cannot change their talents.			0.62
(6) If students work hard in any subject, they will be better at it.			0.54

We have reported both absolute (RMSEA) and incremental fit indices (CFI, IFI); (Hu and Bentler, 1999; Hulpia et al., 2009) to examine the validity of the measures. As per Table 1, neuroplasticity showed a good fit considering both types of fit indices. For RMSEA a value <0.08 explains a reasonable model fit (Musek, 2007), and more strictly values <0.06 shows a good model fit (Hu and Bentler, 1999). Considering incremental fit indices, it is generally suggested that a value close to 0.90 or above indicates a good model fit (Hulpia et al., 2009). For mindset and epistemological belief system, CFI and IFI indicated a good fit, however RMSEA for both measures resulted in a poor fit. Lai and Green (2016) proved that such “inconsistency is not diagnostic of particular problems in model specification or data. Instead, it arises because (a) the two indices, by design, evaluate fit from different perspectives; (b) cutoff values are needed and are being (rightly or wrongly) used, and (c) the meaning of “good fit” and how it relates to fit indices are not well understood in the current literature” (p.234). The Cronbach alpha of the three measures was above 0.70, indicating a good reliability (Table 2).

**Table 2:** Construct validity and reliability of the measures.

Variables	Construct validity				Reliability
	CMIN/DF	CFI	IFI	RMESA	*α*
Neuroplasticity	3.01	0.94	0.94	0.07	0.77
Mindset	5.80	0.939	0.94	0.11	0.79
Epistemological belief system	6.89	0.94	0.94	0.13	0.85

### Data analysis

The data were analyzed using AMOS and SPSS version 24.0 for Windows. Hierarchical regression was conducted to examine the effects of teachers’ neuroplasticity knowledge and mindset (independent variables) on the epistemological belief system (dependent variable) while controlling the effects of age, gender, and years of teaching (background variables). Such an analysis helped us make sure that the claims that explained the structural relationships between independent and dependent variables are epistemologically valid. In social science research, this is called epistemological, ontological, and methodological consistency (Creswell, 2003). Structural equation modeling (SEM) was then performed to examine the effect of teachers’ neuroplasticity knowledge (seven indicators or observed variables) on their epistemological belief system (four dimensions) mediating by mindset (five indicators). Therefore, the final model consisted of three latent variables and 16 observed variables. We used SEM because it assesses “the measurement model (how well the measured variables define their respective construct) and structural model (how well the latent constructs relate to each other) simultaneously” (Meyers et al., 2006, p. 637). In the next step, we examined how teachers with sophisticated/naïve beliefs and growth/fixed mindsets were distributed within the status of good/poor knowledge of neuroplasticity. Therefore, all three variables were recoded into two categories, and the cutoff point to divide each scale was 5% trimmed mean. As the results, the cutoff point means were 3.01 for neuroplasticity, 2.33 for mindset, and 2.24 for epistemological beliefs. In other words, teachers with scores below 3.01 were labeled with poor knowledge of neuroplasticity, 2.33 growth mindset, and 2.24 sophisticated beliefs system.

## Results

### Background variable analysis

Hierarchical regression analysis showed the background variables did not explain a significant variance in teachers’ epistemological belief systems [*F* (3, 341) =0.85, *p* = 0.47, *R*^2^ = −0.00]. As per Table 3, in the first step, the background variables entered the model, which accounted for 0.001 variances (*R*^2^ = 0.001). The regression coefficients for all background variables were not statistically significant. However, the main independent variables (mindset and neuroplasticity) explained a significant variance in teachers’ epistemological belief system [*F* (3,341) = 92.42, *p* < 0.01, *R*^2adj^ = 057]. The regression coefficients for mindset (*β* = 0.35, *p* < 0.01) and neuroplasticity (*β* = −0.52, *p* < 0.01) were statistically significant. Table 3 shows the results of the regression analysis.

**Table 3:** The regression analysis of the background and main variables.

Model	Variables	*R*	*R* ^2^	*R* ^2adj^	*F*	*B*	*β*	*T*	Sig
1	Teaching	0.086	0.007	−0.001	0.85	−0.004	−0.005	0.077	0.938
	Age					0.042	0.052	0.876	0.381
	Gender					0.055	0.063	1.049	0.295
2	Teaching	0.77	0.59	0.59	98.74	0.034	0.041	−0.848	0.397
	Age					0.002	0.002	−0.225	0.822
	Gender					0.028	0.032	1.004	0.316
	Mindset					0.214	0.35	8.69	0.000
	Neuroplasticity					−0.462	−0.52	−12.78	0.000

Dependent variable: teachers’ epistemological beliefs.

The results of the regression analysis suggested that background variables of age, gender, and years of teaching had no significant effects on teachers’ epistemological beliefs; thus, we proceed to the main analysis to test the main hypothesis promoted in this paper.

### Structural equation modeling

Using SEM, we tested this hypothesis: Teachers with correct knowledge of neuroplasticity have more sophisticated epistemological beliefs, mediating by mindset. Considering the following fit indexes, chi-square test, comparative fit index (CFI), incremental fit index (IFI), and mean square of approximation error (RMSEA), the hypothesized model was evaluated. The results produced acceptable overall goodness of fit index (CMIN/df = 2.91; Hoyle and Isherwood, 2013). In addition, the CFI (0.91), IFI (0.91), and RMSEA (0.07) yielded good indexes (Hu and Bentler, 1999; Musek, 2007; Hulpia et al., 2009). These indexes indicate that the hypothesized model fits the observed data.

Analyzing the regression coefficients, the results showed that the path coefficients (direct effects) from teachers’ knowledge of neuroplasticity to their mindset (*β* = −0.69, *p* < 0.01) and epistemological belief system on learning (*β* = −0.72, *p* < 0.01) were statistically significant. Generally, this means that teachers with a higher score in neuroplasticity have a growth mindset and sophisticated epistemological belief system. In other words, with one standard deviation increase in teachers’ knowledge of neuroplasticity, 0.68 and 0.69 standard deviations of their fixed mindset and naïve epistemological belief system decrease, respectively. The path coefficient (direct effect) from mindset to epistemological belief system (*β* = 0.24, *p* < 0.01) shows teachers with higher scores in mindset (fixed mindset) are more likely to fall into the category of naïve epistemological beliefs, meaning that with one standard deviation increase in teachers’ fixed mindset, their naïve epistemological belief system increase by 0.24 standard deviation.

Teachers’ knowledge of neuroplasticity had an indirect effect of −0.17 on their epistemological belief system mediated by mindset. The total effects for all tested paths confirmed the same trend; however, the total effect of the path coefficient from neuroplasticity to the epistemological belief system was larger (−0.88) than the direct effect (−0.72). Table 4 shows the path coefficients of the model.

**Table 4:** The path coefficients of the main variables in the model.

Variable effects in the model	*β* (total effect)	*β* (Direct)	*β* (indirect)	Sig
Neuroplasticity on epistemological belief system	−0.88	−0.72		0.000
Neuroplasticity on mindset	−0.69	−0.69		0.000
Neuroplasticity on epistemological belief system *via* mindset			−0.17	0.000
Mindset on the epistemological belief system	0.24	0.24		0.000

**Figure 2:**
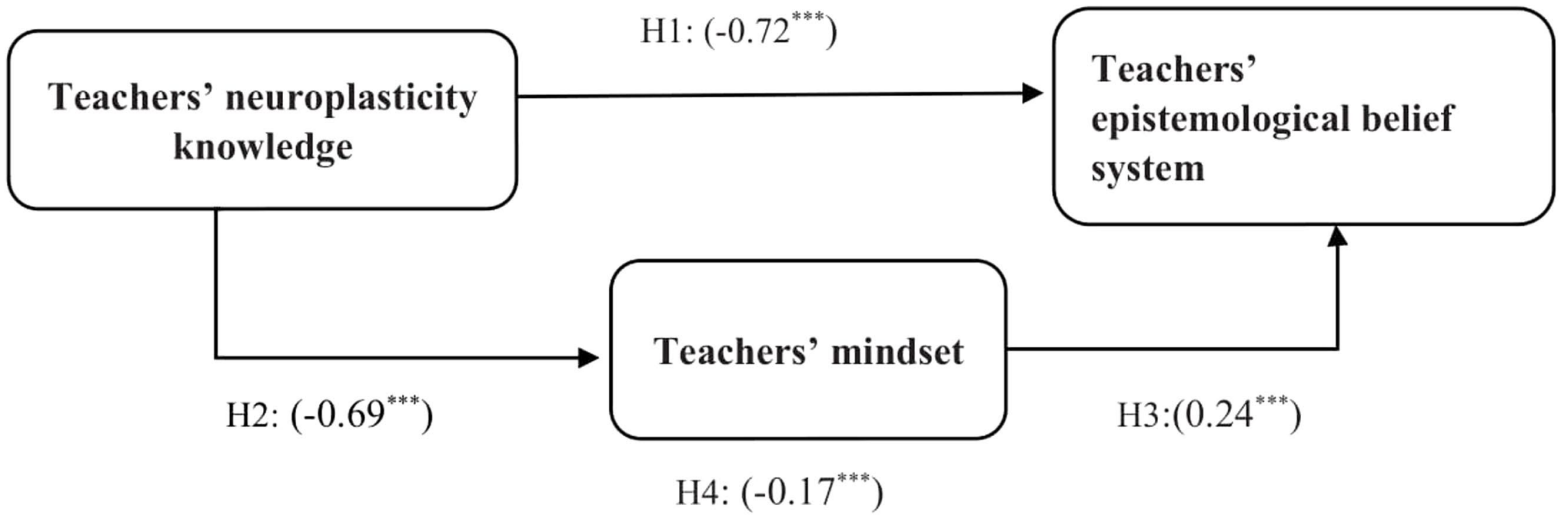
The empirical model of teachers’ neuroplasticity knowledge, mindset, and epistemological belief system. **significant level.

To evaluate the accuracy of the prediction in the structural equations, we examined the proportion of the variance (*R*^2^) accounted for endogenous variables. The amount of variance accounted for mindset was (*R*^2^ = 0.47) and for the epistemological belief system was (*R*^2^ = 0.81). These accounted variances are strong enough in educational sciences (Meyers et al., 2006), suggesting a significant contribution to the literature since this model was examined for the first time. Figure 2 shows the final model developed in this research.

### Descriptive distribution of variables

As found, teachers’ knowledge of neuroplasticity had significant effects on their epistemological belief system and mindset. Descriptive statistics confirmed the same effects. The data analysis showed that 63.4% of teachers with good (correct) knowledge of neuroplasticity were found to have a growth and 36.6% a fixed mindset. In addition, 70% of teachers with good knowledge of neuroplasticity were found to have a sophisticated and 30% a naïve epistemological belief system. The chi-square tests for mindset [*χ*^2^ (df = 1, 21.26) *p* < 01] and epistemological belief system [*χ*^2^ (df = 1, 48.70) *p* < 0.01] showed that these results were statistically significant. Table 5 shows more details about the distribution of the teachers’ knowledge of neuroplasticity within their mindset and epistemological belief system.

**Table 5:** Distribution of teachers’ mindset and epistemological belief system within the knowledge of neuroplasticity.

Epistemological beliefs		Sophisticated beliefs	Naïve beliefs	Total within neuroplasticity
Knowledge of neuroplasticity	Poor knowledge	48	103	151
		31.8%	68.2%	43.8%
	Good knowledge	135	59	194
		70%	30%	56.2%
Total within the epistemological belief system		183	162	345
		53%	47%	100.0%
**Mindset**		**Growth mindset**	**Fixed mindset**	
Knowledge of neuroplasticity	Poor knowledge	58	93	156
		38.4%	61.6%	43.8%
	Good knowledge	123	71	194
		63.4%	37.6%	56.2%
Total within mindset		181	164	345
		52.5%	47.5%	100%

## Discussion

Rooted in the existing literature, we posed four hypotheses to examine the structural relationships among teachers’ knowledge of neuroplasticity, their epistemological belief system, and their mindset. H1 and H2 examined the direct effects of teachers’ knowledge of neuroplasticity on their epistemological beliefs system and mindset. With H1, we stated that teachers’ knowledge of neuroplasticity reduces their naïve epistemological beliefs and with H2, we supposed that teachers’ knowledge of neuroplasticity decreases fixed mindset. The results showed that both hypotheses were supported by our statistical analysis. All fit indexes suggested that the model was empirically acceptable, thus fitting the observed data. The path coefficients from teachers’ knowledge of neuroplasticity to their epistemological belief system (*β* = −0.72, *p* < 0.01) and mindset (*β* = −0.69, *p* < 0.01) were statistically significant and practically strong. This proved that teachers with correct knowledge of neuroplasticity fall less likely into the categories of a naïve epistemological belief system and a fixed mindset. The existing literature also supports this finding. The results of other studies support that teachers with genuine or scientific knowledge, particularly knowledge about the brain or educational neuroscience, are less likely to have a poor belief system and neuromyths (Dubinsky et al., 2013; Ferrero et al., 2016; Wilcox et al., 2021; Ferreira and Rodríguez, 2022). Teachers’ fixed mindset, naïve epistemological beliefs, and neuromyths all constitute a teacher poor belief system that may hinder the quality of their pedagogical skills and decisions.

H3 and H4 were formulated to examine the effects of teachers’ mindset on their epistemological belief system. With H3, we tested the direct effect of teachers’ mindset stating that teachers with a growth mindset have less likely a naïve epistemological belief system. Through H4, we posed that teachers’ mindset mediates the negative relationship between knowledge of neuroplasticity and the epistemological belief system. The results of the data analysis significantly supported both hypotheses. The path coefficient (direct effect) from mindset to epistemological belief system (*β* = 0.24, *p* < 0.01). This indicates when teachers have a growth mindset, they are more likely to grasp a more sophisticated belief system and vice versa. The indirect effect of teachers’ knowledge of neuroplasticity on their epistemological belief system *via* mindset was −0.17. These findings are in line with the current literature. Multiple empirical research has suggested that teachers and students with a growth mindset, show more sophisticated beliefs and effective actions and characters (Aronson et al., 2002; Blackwell et al., 2007; Rissanen et al., 2018a,b, 2019, 2021; DeLuca et al., 2019; Dweck and Yeager, 2019; Burnette et al., 2020; Yeager and Dweck, 2020; Wang et al., 2021). These findings show that teachers’ mindset has a significant effect on their pedagogical thinking, decisions, and actions toward students.

We further did a descriptive analysis of data to study how teachers with correct (good) and incorrect (poor) knowledge of neuroplasticity distributed across mindset and epistemological belief system. The results proved the same trend as discussed above. In other words, teachers with correct knowledge of neuroplasticity were mostly distributed across sophisticated beliefs and growth mindset. However, 36.6% and 30% of teachers with good knowledge of neuroplasticity were found to have a fixed mindset and a naïve epistemological belief system, respectively. One reason might be due to methodological issues. In quantitative research, when data are collected by a survey with different statements, participants might have a wrong perception of statements. The other reason could be related to the general belief system of the participants rooted in their social and cultural background. When teachers have a strong personal belief system, they may resist against scientific facts and reject integrating them into their pedagogical decisions.

## Implication

### Theoretical application

In line with the existing literature discussed, we agree that teachers’ knowledge of neuroplasticity, epistemological belief system, and mindset are all important variables that have significant effects on their pedagogical practice. However, in most previous studies, the epistemological belief system was examined as a predictor of other traits and performance (e.g., Yilmaz-Tuzun and Topcu, 2008; Demirbag and Bahcivan, 2022). In a very basic study, Schommer (1993) found that students academic achievement were regressed on their epistemological beliefs: The less the students believed in quick learning, the higher the GPA they acquired. Mindset or implicit theory of intelligence was also found to play a predicting role in previous empirical studies (Aronson et al., 2002; Good et al., 2003; Blackwell et al., 2007; Zhang et al., 2019). In many studies, Dweck examined how students’ mindset influences the ways they do different tasks. Yeager and Dweck (2020) reviewed different studies from different contexts and concluded that mindset is a predicting phenomenon for outcome and achievement. In the present research, we argued that these variables have different epistemic positions where teachers’ knowledge of neuroplasticity is superior to mindset and epistemological beliefs. “Knowledge has been typically associated with genuine or scientific cognition that can provide truth whereas belief has been thought to present mere appearances or subjective opinion, usually founded on sense perceptions” (Kim, 2018). In line with the premise that knowledge shall be superior to a personal belief in teaching, we examined a model consisting of teachers’ knowledge and beliefs in which teachers’ knowledge of neuroplasticity was hypothesized to have effects on their mindset and epistemological belief systems. So, in this research we implicitly addressed the following concern and problem in the literature to propose a new and different conceptual model: If epistemological belief system and mindset predict individuals’ performance, then how can we help students and teachers develop a sophisticated epistemological belief system and growth mindset? Based on the results of the present study, promoting teachers’ knowledge of neuroplasticity helps them become practitioners with a more sophisticated epistemological belief system and growth mindset. Therefore, we have theoretically proposed a conceptual hierarchy to explain the epistemic relationship between teachers’ knowledge of neuroplasticity, their mindset, and their epistemological belief system.

### Practical application

Since the 1970s, there has been a cognitive shift in research on teaching, arguing that teachers are no longer the consumers of knowledge produced by university researchers but are in the epistemological position of crafting knowledge for teaching (Connelly and Clandinin, 1985; Elbaz, 1991). The core of this shift was to claim that teachers can develop personal and practical knowing while engaging in teaching (Gholami and Husu, 2010). Teachers’ mindset and epistemological belief system can be considered a significant part of teachers’ personal and practical knowing. Our findings showed that teachers’ knowledge of neuroplasticity may help teachers to develop a sophisticated belief system and growth mindset. So, based on the results of this research, policymakers should integrate neuroplasticity knowledge into in-service teachers’ professional development for supporting and developing teachers’ personal and practical knowing. In addition, based on the results of this study, teacher educators should integrate educational neuroscience as a fundamental dimension of teacher education programs. In addition to pedagogical content knowledge, general pedagogical knowledge, and subject knowledge, knowledge of the brain and neuroplasticity should receive an epistemic identity in teaching studies and the teacher education curriculum.

## Limitations

The present research has two basic limitations. The structural relationship between teachers’ neuroplasticity knowledge, epistemological belief system, and mindset was examined for the first time in this research and a limited educational context. So, the results should be generalized with caution. In addition, we suggest other researchers re-examine or re-design this model for more empirical reliability and validity. We also found that a significant percentage of teachers with correct or good knowledge of neuroplasticity have a fixed mindset and a naïve epistemological belief system. We believe this might be due to teachers’ social and cultural belief systems. Because social and cultural beliefs are a deeper part of teachers’ belief systems, there should be further qualitative research to study why teachers with good knowledge of neuroplasticity still have a fixed mindset or a naïve epistemological belief system.

## Data availability statement

The raw data supporting the conclusions of this article will be made available by the authors, without undue reservation.

## Ethics statement

Ethical review and approval were not required for the study on human participants in accordance with the local legislation and institutional requirements. Written informed consent from the patients/ participants or patients/participants legal guardian/next of kin was not required to participate in this study in accordance with the national legislation and the institutional requirements.

## Author contributions

All authors listed have made a substantial, direct, and intellectual contribution to the work and approved it for publication.

## Conflict of interest

The authors declare that the research was conducted in the absence of any commercial or financial relationships that could be construed as a potential conflict of interest.

## Publisher’s note

All claims expressed in this article are solely those of the authors and do not necessarily represent those of their affiliated organizations, or those of the publisher, the editors and the reviewers. Any product that may be evaluated in this article, or claim that may be made by its manufacturer, is not guaranteed or endorsed by the publisher.

## Supplementary material

The Supplementary material for this article can be found online at: https://www.frontiersin.org/articles/10.3389/fpsyg.2022.1042891/full#supplementary-material
