# Supplementary material for: Empirical model of teachers’ neuroplasticity knowledge, mindset, and epistemological belief system
Source: Front Psychol. 2022 Dec 8;13:1042891. doi: 10.3389/fpsyg.2022.1042891 (PMC9773884; doi:10.3389/fpsyg.2022.1042891)
Supplement: Supplementary file 1 [file Presentation_1.pdf]

Appendixes

Neuroplasticity Items

1) Learning occurs through the modification of the brain's neural connections (C).

2) Extended rehearsal of some mental processes can change the shape and structure of some parts of the brain (C).

3) Mental capacity is hereditary and cannot be changed by the environment or experience (I).

4)There are sensitive periods in childhood when it is easier to learn things (I).

5) Learning problems associated with developmental differences in brain function cannot be remediated by education (I).

6) There are critical periods in childhood after which certain things can no longer be learned (I).

7) Normal development of the human brain involves the birth and death of brain cells (C).

8) Production of new connections in the brain can continue into old age (C).

9) Vigorous exercise can improve mental function (C).

Mindset Items

1) Students' intelligence is something very basic about them that they cannot change very much.

2) No matter how much intelligence students have, they can always change it quite a bit.

3) Students may learn new things, but they cannot change their intelligence.

4) Students have a certain talent in certain subjects (e.g., math, sports), and they cannot change it.

5) Students can learn new things, but they cannot change their talents.

6) If students work hard in any subject, they will be better at it.

Epistemological Belief Items

Fixed Ability

1) The ability to learn is innate.

2) Genius is 10% ability and 90% hard work.

3) Going over and over a difficult textbook chapter does not usually help you understand it.

4) Almost all the information you will get from a textbook you will learn during the first reading.

5) Some people are born good learners, and others are stuck with limited abilities.

6) The really smart students do not need to work hard to do well in school.

Simple Knowledge

7) Most words have one clear meaning.

8) When I study, I look for specific facts.

9) Learning definitions word by word is always necessary to do well in tests.

10) I do not like movies that do not have an ending.

11) I appreciate teachers who have very clear and well-organized instructions and stick to their plans.

12) A really good way to understand a textbook is to reorganize the information according to your scheme.

13) Being a good student involves memorizing facts.

Quick Learning

14) Good students understand things quickly.

15) If a person cannot understand something within a short amount of time, they should keep on trying.

16) Learning is a slow process of building knowledge.

17) I often wonder how fast smart students learn.

18) Working hard on a difficult problem for a long time only pays off for smart students.

Certain Knowledge

19) The only certain thing is uncertainty itself.

20) Truth is unchanging.

21) Nothing is certain but death and taxes.

22) Today’s facts may be tomorrow’s fiction.

23) If scientists try hard enough, they can find the truth in everything.

24) Scientists can ultimately get the truth
